# Insights into the LiMn_2_O_4_ Cathode Stability in Aqueous Electrolytes

**DOI:** 10.1021/acs.chemmater.4c00888

**Published:** 2024-06-03

**Authors:** Juan Carlos Gonzalez-Rosillo, Maxim Guc, Maciej Oskar Liedke, Maik Butterling, Ahmed G. Attallah, Eric Hirschmann, Andreas Wagner, Victor Izquierdo-Roca, Federico Baiutti, Alex Morata, Albert Tarancón

**Affiliations:** † Catalonia Institute for Energy Research (IREC), Jardins de les Dones de Negre 1, Planta 2, 08930 Sant Adrià del Besòs, Barcelona, Spain; ‡ Catalan Institution for Research and Advanced Studies (ICREA), Passeig Lluís Companys 23, 08010 Barcelona, Spain; § Institute of Radiation Physics, Helmholtz-Zentrum Dresden − Rossendorf, Bautzner Landstraße 400, 01328 Dresden, Germany

## Abstract

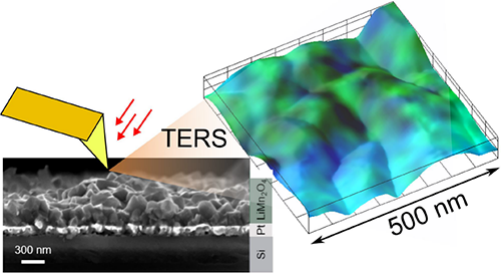

LiMn_2_O_4_ (LMO) cathodes present large stability when cycled in aqueous electrolytes, contrasting with their behavior in conventional organic electrolytes in lithium-ion batteries (LIBs). To elucidate the mechanisms underlying this distinctive behavior, we employ unconventional characterization techniques, including variable energy positron annihilation lifetime spectroscopy (VEPALS), tip-enhanced Raman spectroscopy (TERS), and macro-Raman spectroscopy (with tens of μm-size laser spot). These still rather unexplored techniques in the battery field provide complementary information across different length scales, revealing previously hidden features. VEPALS offers atomic-scale insights, uncovering cationic defects and subnanometer pores that tend to collapse with cycling. TERS, operating in the nanometric range at the surface, captured the presence of Mn_3_O_4_ and its dissolution with cycling, elucidating dynamic changes during operation. Additionally, TERS highlights the accumulation of SO_4_^2–^ at grain boundaries. Macro-Raman spectroscopy focuses on the micrometer scale, depicting small changes in the cathode’s long-range order, suggesting a slow but progressive loss of crystalline quality under operation. Integrating these techniques provides a comprehensive assessment of LMO cathode stability in aqueous electrolytes, offering multifaceted insights into phase and defect evolution that can help to rationalize the origin of such stability when compared with conventional organic electrolytes. Our findings advance the understanding of LMO behavior in aqueous environments and provide guidelines for its development for next-generation LIBs.

## Introduction

The development of advanced cathode materials for lithium-ion batteries (LIBs) has been at the forefront of research aiming at improving the performance, cost, and safety of energy storage systems. Since its first reports by Hunter and Goodenough’s group back in the 80s,^1,2^ there has been a continuous and growing interest from both academic and industrial perspectives in LiMn_2_O_4_ (LMO), which has led to a deep understanding of its structure, properties, advantages, and challenges.^3^ LMO belongs to the family of cubic spinel oxides (*Fd*3*m* space group), in which Li is tetrahedrally coordinated and Mn is octahedrally coordinated, which allows for 3D Li^+^ diffusion channels that could potentially lead to fast-charging cathodes, as shown, for instance, in other spinel-type materials.^4^

Despite its relatively high theoretical capacity (148 and 296 mAh·g^–1^ for LiMn_2_O_4_ in the regular and extended cycling ranges, respectively), there are plenty of reports showing concerns regarding the long-term stability when cycled in conventional organic electrolytes. In particular, Mn dissolution has been claimed to be the main drawback of this material, partially due to Jahn–Teller distortions and Mn disproportionation.^5^ However, other effects play a role since Mn dissolution also occurs in the fully charged state,^6,7^ unexpected if disproportionation would be the only responsible of Mn^2+^ dissolution. Recent works have shown with great detail the degradation mechanism in LiMn_2_O_4_ in LIBs through advanced characterization techniques, as excellently summarized by Prof. Ju Li’s team in their review.^3^ Essentially, these recent studies highlighted significant surface-related phenomena. These observations include Mn reduction during charging and Mn oxidation during discharging at the surface, oxygen loss linked to Mn reduction, and surface reconstruction upon charging.^8−11^ This ultimately results in Mn dissolution and surface phase transformations, contributing to capacity decay and increased impedance.^12^ In addition, advanced characterization using in situ techniques has revealed the formation of Mn_3_O_4_ at high charge voltages of 4.2 V and Li_2_Mn_2_O_4_ at discharge voltages of 3.3 V. These electrochemically induced phase transformations are partially irreversible and can lead to particle cracking during cycling, further exacerbating the reactive surface area.^13^

Interestingly, despite the intricate challenges posed by surface-related phenomena and structural instability in LiMn_2_O_4_ when employed with conventional organic electrolytes, a remarkable shift occurs when this cathode material is cycled in aqueous electrolytes. In this alternative environment, LiMn_2_O_4_ exhibits a surprising level of stability, seemingly untouched by the issues that affect its performance in organic electrolytes, besides the well-known Mn dissolution.^14−19^ This stark contrast in behavior underscores the need for a thorough investigation into the underlying mechanisms responsible for this stability divergence, setting the stage for our exploration of LMO’s performance in aqueous environments using unconventional techniques, trying to ask a simple question: why is LMO more stable in aqueous electrolytes? Can we learn anything from the study of high-stability operation in an aqueous electrolyte that can be extrapolated to organic electrolytes?

In this work, we examine the structural and phase evolution of LMO films in an aqueous electrolyte as a function of cycling by three novel characterization techniques that allow us to examine surface and bulk properties at very different length scales: tip-enhanced Raman spectroscopy (TERS), macro-Raman spectroscopy, and variable energy positron annihilation lifetime spectroscopy (VEPALS).

TERS is a relatively new surface-sensitivity technique that overcomes the spatial resolution limitations of conventional Raman (∼500 to 1000 nm). For that, TERS combines the spectroscopic power of Raman spectroscopy with the spatial resolution of a scanning probe microscope. This is achieved by carefully focusing the laser at the apex of a tip coated with a thin metal able to generate surface plasmon resonances and lightning-rod effect to enhance the Raman signal.^20^ This enhancement has led even to detect single molecules.^21−24^ While it is widely used to study organic materials, its application to ceramic materials is not yet widespread. For instance, the use of TERS in batteries is still in its infancy with only few papers using the technique in the field among the thousands of battery-related research articles published every year.^25−27^

With a completely different and complementary approach, macro-Raman spectroscopy uses a very large laser spot size (in our case. 50 μm in diameter) and ultralow energy densities (tens of W·cm^–2^ vs tens of kW·cm^–2^ in conventional Raman) to ensure no influence on the sample while being sensitive to phases located in a true representative area, with the averaged signal from 10^3^ to 10^4^ grains under the measurement spot, considering spherical size grains of ∼150 to 200 nm as those in our films.^28^

In VEPALS, the films are irradiated with positrons that recombine (annihilate) with electrons in the films at defined depths, creating γ rays, which are subsequently detected.^29^ The measurement of positron lifetime provides direct information on the type and size of negatively charged and neutral defects through their characteristic time before encountering an electron, whereas pores are characterized based on the lifetime of residing and bouncing in them a bound state of positron and electron, the so-called Positronium (Ps).^30,31^ The concentrations of positrons and Ps are evaluated through the relative intensities of the different lifetime components. In the context of battery research, VEPALS has found notable applications, particularly in studies involving layered compounds like LiCoO_2_. In such cases, positron lifetime has been proposed as a reliable indicator of the lithiation state of grain boundaries.^32^ Furthermore, in investigations of compounds like LiNi_1/3_Mn_1/3_Co_1/3_O_2_, operando experiments have revealed a gradual increase in divacancy and vacancy agglomerate formation during the charging process, culminating in the transformation of these agglomerates into one-dimensional vacancy chains as the charge cycle nears completion.^33^ These findings underscore the versatility and significance of VEPALS in understanding complex defects and phenomena within battery materials.

These three complementary techniques, rarely explored within the battery field, offer a multifaceted perspective on phase and defect evolution within LMO cathodes and their performance stability in aqueous electrolytes. Operating across a broad spectrum of length scales, they collectively reveal previously unseen features. Notably, VEPALS exposes the existence of cationic defects and subnanometer pores, providing insights at the atomic scale of the dynamic changes with cycling. TERS captures the presence of Mn_3_O_4_ at the surface and its subsequent dissolution during cycling with a resolution better than 20 nm. Simultaneously, TERS also highlights the accumulation and potential incorporation of SO_4_^2–^ ions, primarily at grain boundaries, while macro-Raman spectroscopy focuses on the material’s long-range order, depicting small changes at the micrometer scale. Our work contributes to a more profound understanding of the stability of LMO in aqueous electrolytes with the hope that this knowledge can be extrapolated to implement solutions toward longer stability in conventional organic electrolytes.

## Results and Discussion

### Characterization of the As-Deposited LMO Films

**Figure 1:**
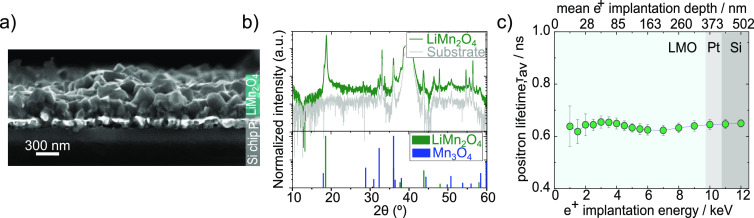
Characterization of the as-deposited films. (a) SEM cross-section of the as-deposited LMO films. (b) XRD pattern of the LMO films and the bare Pt-coated Si substrate. (c) Positron lifetime vs implantation energy.

LMO thin films were fabricated by large area pulsed laser deposition with our well-established method of compensating Li losses with a multilayer deposition of LiMn_2_O_4_ parent compound and Li_2_O acting as Li reservoir.^17,34−38^ When grown on top of Pt-coated Si chips, this method produces relatively rough polycrystalline layers with high phase purity. For this experiment, a batch of 300 nm thick LMO films was produced (for details, see the Experimental section). SEM shows a cross-section of the layers that are dense and rough (as expected) with well-connected grains with sizes of 150–300 nm (Figure 1a). In line with our previous results,^17,37^ the XRD patterns of the layer evidence the polycrystalline nature of our films with small quantities of Mn_3_O_4_ impurities. Despite the difficulty of performing more sophisticated approaches (Rietveld refinement) in these data, one can see, for instance, that the most intense line that can be unambiguously assigned to Mn_3_O_4_ (2θ = 28.91°, *I* ≈ 40%) is almost not visible in the diffractogram. In contrast, the (400) reflection of LMO (2θ = 43.87°, *I* ≈ 33%) is identified with large intensity.

We further characterized the films with VEPALS. The average positron lifetime versus positron implantation energy is quite homogeneous along the film thickness (Figure 1c). The deconvolution of the spectra (see ref (37), experimental methods and Supporting Information, Section I) showed four main types of defects: small vacancy-like defects and their clusters and two families of subnm pores with spherical sizes *d*_3_ ≈ 0.47 nm and *d*_4_ ≈ 0.75 nm (calculated based on^39^). Overall, both pore families and vacancy-related defects exhibit homogeneous distributions across the thickness of the film. Comparing the relative intensities, one can see that the vacancy-related defects dominate the average lifetime of the positrons, indicating a larger presence of vacancies than pores. These kinds of vacancies have also been observed by PALS in LiCoO_2_ cathodes and have been ascribed to lithium vacancies and clusters of lithium vacancies, respectively.^29,32^ However, we would rather be cautious over the assignment of the type of vacancy; see Supporting Information, Section I for this discussion. Overall, these PALS measurements demonstrate the presence of cationic defects and pores in our films, which potentially play a role in suppressing and/or alleviating commonly observed Jahn–Teller distortions in LMO.^37,40^

**Figure 2:**
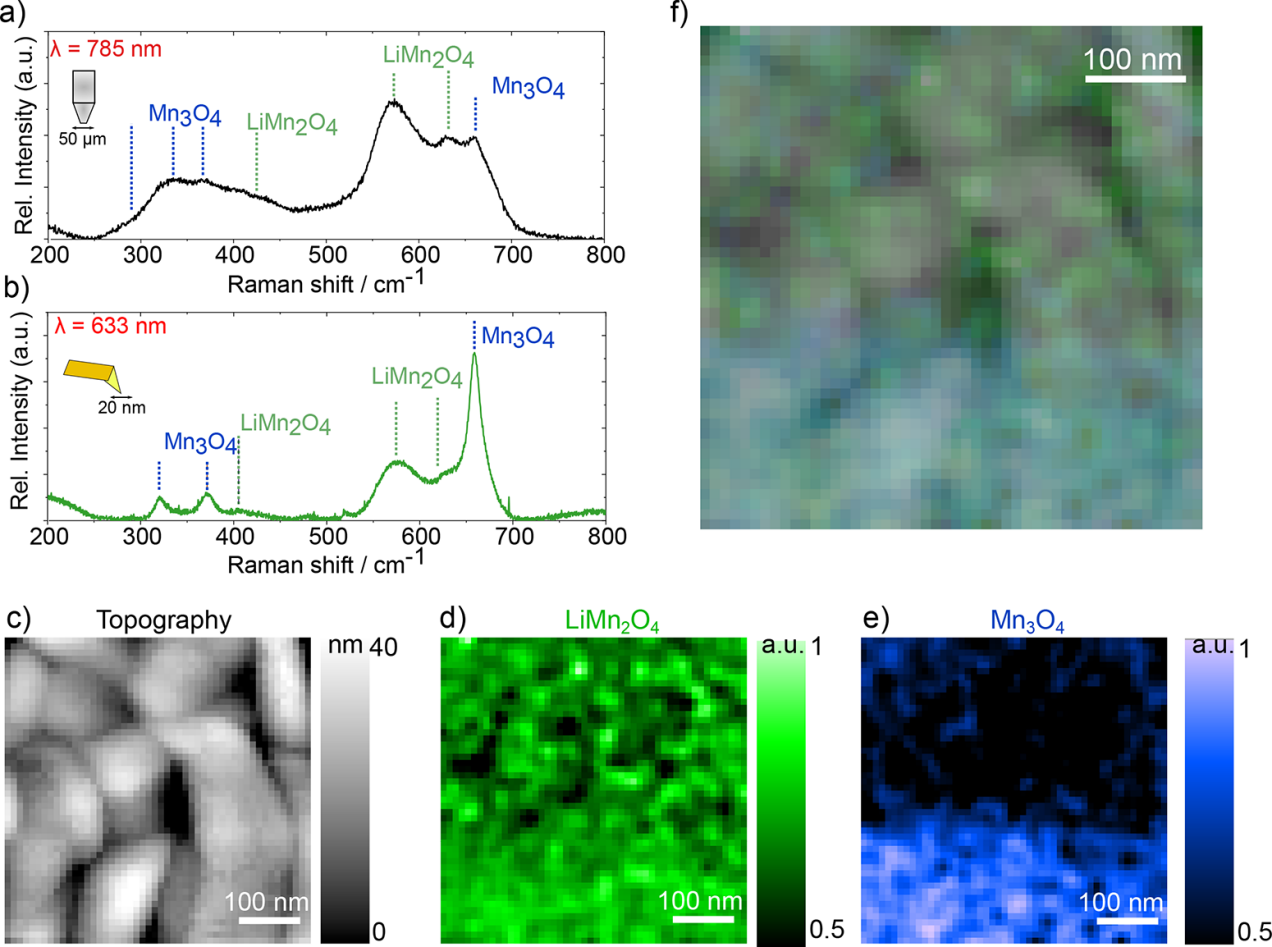
Raman spectroscopy and electrochemistry. (a) Macro-Raman spectroscopy. (b) Average TERS spectrum of the measurement. (c) Topography obtained during the TERS mapping. Size 500 × 500 nm^2^ (d) TERS map, 500 × 500 nm^2^. Green represents the LiMn_2_O_4_ phase and (e) blue the Mn_3_O_4_ phase. (f) Overlay of topography and TERS, 500 × 500 nm^2^.

Regarding the Raman characterization of the films, existing differences between the spectra obtained with macro-Raman spectroscopy and TERS highlight important differences between the bulk and the surface. Please note that peak assignment and details on spectra treatment are detailed in the Supporting Information, Section III. On one hand, the macro-Raman spectroscopy of the as-deposited state films included in Figure 2a shows the presence of mainly LiMn_2_O_4_ and impurities of Mn_3_O_4_,^41,42^ in agreement with the XRD results. In addition, the presence of off-stoichiometric Li_*x*_Mn_2_O_4_ phase cannot be discarded from the measured spectra due to the shoulder appearing at the LiMn_2_O_4_ band,^41^ suggesting the presence of vacancy defects in the bulk of the films, as suggested by PALS. On the other hand, TERS measurements with higher surface sensitivity complement this information by assigning a large presence of Mn_3_O_4_ at the surface and subsurface level. This is clearly shown in the average TERS spectra corresponding to a 500 × 500 nm^2^ spectroscopy scan, Figure 2b, where the main peak of Mn_3_O_4_ (located at ∼660 cm^–1^) is now more intense than the LiMn_2_O_4_ peaks, in stark contrast with the macro-Raman spectroscopy measurements. The topography acquired during the TERS measurement, Figure 2c, shows several grains with very distinguishable grain boundaries. The TERS maps representing the relative intensity across the map of the LiMn_2_O_4_ phase, Figure 2d, and the Mn_3_O_4_ phase, Figure 2e show a clear contrast. While the LiM_2_O_4_ is more homogeneously distributed, the Mn_3_O_4_ phase seems to be located more at the bottom part of the image. The full power of the technique is revealed by superimposing the topography and the spectroscopy maps, Figure 2f. Here, one can see that LiMn_2_O_4_ dominates the grains. In contrast, Mn_3_O_4_ is clearly present at the grain boundary level (all across the image) and extensively in some particular grains. For more details about how these maps are built, the demonstration of the TERS effect on our setup, and an enlarged version of the maps, the reader is referred to the Supporting Information, Section III.

Combining the different techniques, one can conclude that the LMO films are composed mainly of the electrochemically active spinel phase and impurities of Mn_3_O_4_, which are located primarily at the surface of the films and especially at the grain boundary level. The films also show the presence of vacancy-like defects and their clusters (probably at grain boundaries) across the film and to a lesser extent, two families of subnm pores.

### Characterization of the LMO Films during Cycling

**Figure 3:**
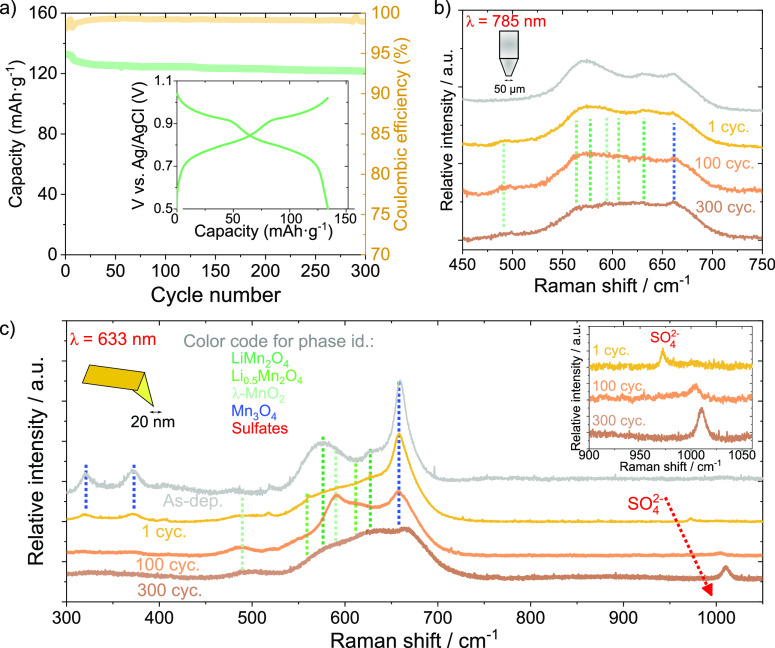
Raman spectroscopy of the cycled films. (a) Electrochemical cycling of the LMO films. The inset shows an example of charge–discharge profile (cycle #50) (b) Macro-Raman spectroscopy. (c) Average TERS average spectra for each film. Inset shows a zoomed-in view of the sulfate-band region. In (b) and (c), the as-deposited is plotted for comparison.

The fabricated polycrystalline LMO films were cycled as previously detailed elsewhere showing the same electrochemical and structural properties as those previously reported by our group.^17,37,43^ Upon cycling in a sulfate-based aqueous electrolyte (see Experimental Section for details), the films show the expected two voltage plateaus and capacities of ∼126 mAh·g^–1^ after 300 cycles, with a Coulombic efficiency >99% (Figure 3a and inset). The electrochemical properties also evidence that the films are composed mainly of electrochemically active LiMn_2_O_4_ since its capacity approaches the practical capacity of LiMn_2_O_4_ that is commonly found in literature.^3^

We proceed here with a comprehensive exploration of the Raman signature evolution within our LMO films throughout the cycling process. Specifically, we analyzed films at nominally 100% state-of-charge and SoC (i.e., discharged state) at different stages of cycling: After 1, 100, and 300 cycles, macro-Raman spectroscopy allows us to scrutinize these changes. Figure 3b illustrates a noteworthy trend: the primary peaks of the electrochemically active phases exhibit a relative intensity decrease concerning Mn_3_O_4_ inactive impurities (Figure S5a in the Supporting Information). It is essential to note that our macroscopic Raman spectroscopy approach, given its true average nature, is expected to detect all electrochemically active phases—namely, LiMn_2_O_4_, Li_0.5_Mn_2_O_4_, and λ-MnO_2_. This detection remains consistent with literature findings as our capacities stay in the expected range of the practical capacity of the material.^3^ This implies that a substantial portion of Li^+^ ions resides within the lattice as LiMn_2_O_4_, even when the material is nominally at 100% State of Charge (SoC), as detected by our macro-Raman spectroscopy measurements and other studies in the field.^44^ The observed smearing and decrease of the intensity of the electrochemically active Raman modes (Figure S5 in the Supporting Information) could imply a gradual increase in film disorder and/or decrease of the crystalline quality. This phenomenon correlates with the rise in vacancy-like defects and their clusters, as identified through PALS in the next section. The enhanced disorder and reduced crystalline quality within electrochemically active phases during cycling may ultimately govern the outstanding long-term stability and high Coulombic efficiency of these films in aqueous electrolytes.

In contrast, the main mode of Mn_3_O_4_ appears to remain largely unaffected at the macroscopic level, emphasizing its inactive electrochemical role. However, a closer examination of the surface using TERS mapping during cycling unveils two critical observations. Figure 3c compares the average TERS spectra extracted from the corresponding maps. First, there is a significant decrease in the Mn_3_O_4_ signal at the surface with cycling (Figure S5b). This intriguing observation hints at the partial dissolution of these impurities, considering the presence of Mn^2+^ within their structure.^3,45,46^ Please note that we do not observe any obvious phase appearance that would suggest a transformation rather than the dissolution of Mn_3_O_4_ at the surface. The observation of potential Mn_3_O_4_ dissolution in the sulfate-based electrolyte employed here is in stark contrast with recent evidence of Mn_3_O_4_ formation in nitrate-based electrolytes.^47^ Second, the sulfate ion signal exhibits increased intensity and a blue shift, evolving from ∼972 cm^–1^ after 1 cycle to ∼1010 cm^–1^ after 300 cycles. This shift suggests a transition from absorbed SO_4_^2–^ ions to a scenario in which these adsorbates form shorter and stronger bonds with the film. It might even be possible that Li_2_SO_4_ is actually formed as a protective and beneficial SEI at the surface and interfaces (grain boundaries), given that the main band of solid Li_2_SO_4_ is reported to be precisely at 1010 cm^–1^. However, simulations are needed to confirm this point from a theoretical perspective.

**Figure 4:**
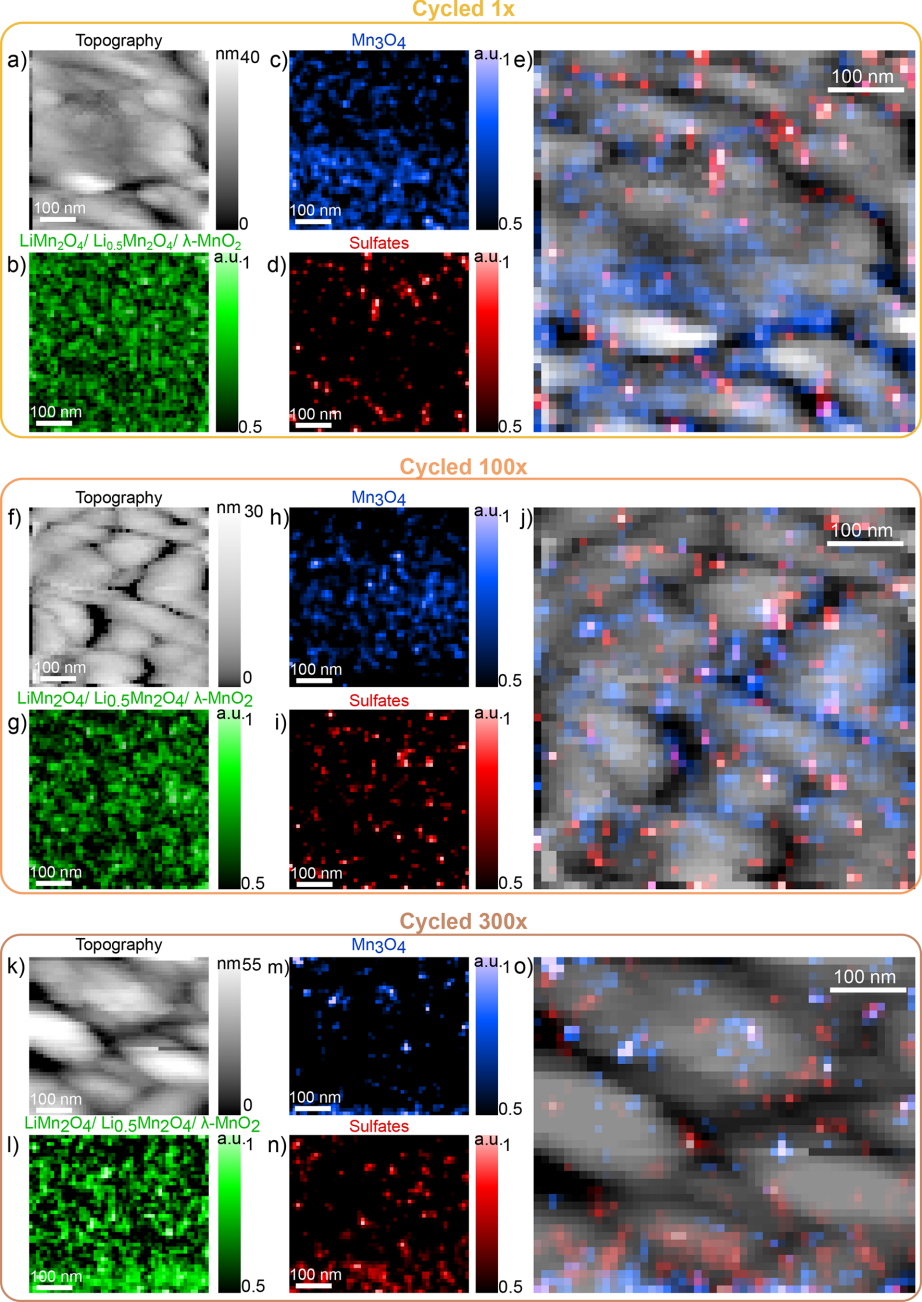
TERS maps. For the film cycled 1 time, size 500 × 500 nm^2^: (a) topography, (b–e) TERS maps of the electrochemically active, Mn_3_O_4_, sulfate band and overlay, respectively. For the film cycled 100 times, size 500 × 500 nm^2^: (f) Topography, (g–j) TERS maps of the electrochemically active, Mn_3_O_4_, sulfate band, and overlay, respectively. For the film cycled 300 times, size 500 × 440 nm^2^: (k) Topography, (l–o) TERS maps of the electrochemically active, Mn_3_O_4_, sulfate band, and overlay, respectively.

Figure 4 shows the TERS maps and overlays for the cycled films. The reader can find in the Supporting Information, Section III, a larger version of these images and extra overlays. For instance, the reader will find overlays between the electrochemically active phases (green maps in Figure 4) and their corresponding topographies, showing that they are well distributed along the grains. Here, for each panel, we show the TERS maps for the electrochemically active phases (green maps), the Mn_3_O_4_ phase (blue maps), and the sulfate-based band (red maps) as a function of cycling. For facilitating the location, the overlays show the superposition of the topography with the Mn_3_O_4_ (blue) and sulfate-based (red) bands. Comparing the spatial distribution of Mn_3_O_4_ (blue) and sulfate signals (red) in films cycled 1× (Figure 4a–e), 100× (Figure 4f–j) and 300× (Figure 4k–o) reveals a notable trend. The proportion of maps dominated by Mn_3_O_4_ (blue) decreases with cycling, especially compared to that of the as-deposited (Figure 2f). By 300 cycles, the few visible blue spots are confined close to certain grain boundaries. Moreover, both the 100× and 300× cycled maps exhibit the sulfate signal (red) predominantly at grain boundaries, clearly visible in Figure 4j,o for the 100 and 300 cases, respectively.

**Figure 5:**
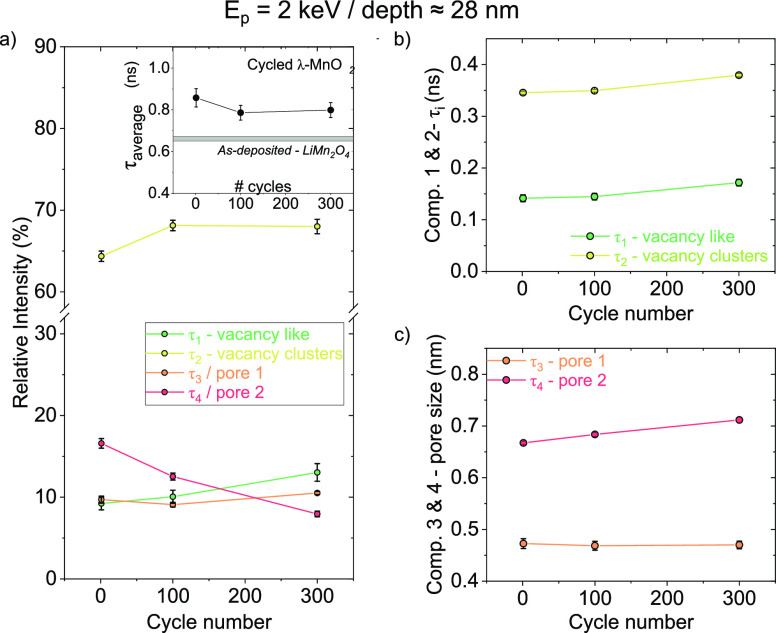
Positron annihilation lifetime spectroscopy of cycled films. (a) Relative intensity of each type of defect as a function of cycling. Inset shows the positron average lifetime. (b) Lifetime for components τ_1_ (vacancy-like defects) and τ_2_ (clusters of vacancies) as a function of size. (c) Pore size derived from τ_3_ and τ_4_, resulting in *d*_3_ and *d*_4_, respectively, as a function of cycling. Error bars are very small in some cases to be seen in the plot.

To complement our findings, VEPALS measurements were carried out to gain insights into defect evolution during cycling. Detailed descriptions of the deconvolution of the PALS signal into various components can be found in the Supporting Information, Section I. A critical observation emerges from the data analysis: our films’ average positron lifetime (τ_average_) decreases from the first cycle onward, stabilizing after 100 and 300 cycles, the inset of Figure 5a.

This decline in τ_average_, directly linked to the free volume, can be attributed to the dynamic evolution of the electrolyte solution within grain boundaries and through pores within the film. Deconvolution of the different defect signals reveals that the most significant changes occur in τ_4_, representing a family of larger subnm pores. Its relative intensity, proportional to pore density, decreases from approximately 17% after 1 cycle to around 8% after 300 cycles, Figure 5a. This decline could imply that a part of these characteristic pores might be filled, likely with sulfate ions, as suggested by our TERS measurements, or the pore size increases by coalescence to some extent. This shift in pore density redistributes the contribution of the other three components within the defects, which tend to become more significant in the signal. Specifically, the smaller vacancies (τ_1_) and their clusters (τ_2_) demonstrate an increase with cycling (Figure 5b). This trend is mirrored in the large family of pores, τ_4_, which increases in size at a rate higher than that of vacancy clusters, Figure 5c. In contrast, the smaller family of pores, τ_3_, appears unaffected by cycling. These changes, primarily the increases in τ_1_, τ_2_, and τ_4_, suggest the dissolution of Mn^2+^ (natively present in the Mn_3_O_4_ structure) into the solution, leaving behind larger defects.^46^ Nevertheless, the dissolution rate in pores is expected to be higher than due to vacancies and their clusters, given the higher free volume, as generally probed by PALS. It also aligns with the observed increase in film disorder, consistent with the smearing out of the Raman bands noted by macro-Raman spectroscopy and TERS. For completeness, it is noteworthy that the substantial reduction in pore density (τ_4_) is even more pronounced at the film’s bottom portion, essentially vanishing progressively with cycling (see Supporting Information, Section II).

Our study aligns with recent research that underscores the dominant role of fast grain boundary and surface diffusion, surpassing grain diffusion by a staggering 4 orders of magnitude.^48^ Mürter et al. convincingly argued that the wide range of reported diffusion coefficients in the literature likely stems from underestimating the contribution of grain boundaries. In our work, we provide experimental evidence of the complex chemistry of grain boundaries during cycling, a critical factor that demands consideration. Notably, Mürter et al. identified residual electrolyte peaks in their X-ray diffraction (XRD) pattern. We propose an intriguing possibility: these residuals may, in fact, become integral parts of the material’s structure after cycling, predominantly within the grain boundaries. This notion suggests that grain boundaries can actively evolve and adapt, potentially absorbing ions from the electrolyte as part of their transformation. These insights emphasize the need to account for the active role of grain boundaries in the design of protective coatings, especially in high-voltage cathodes.

On the other hand, importantly, grain boundaries within LiMn_2_O_4_ are known to carry a positive charge and establish a space charge layer around them.^48,49^ This inherent property makes them susceptible to ion adsorption from the electrolyte during cycling, warranting careful consideration in the protective coating design. Understanding the behavior of grain boundaries in high-voltage cathodes represents a pivotal step toward the development of more sustainable and stable electrode materials.

The surface dilution of Mn_3_O_4_ during cycling, primarily restricted to a few grain boundaries after 300 cycles, stands in stark contrast to its behavior in organic electrolytes. In organic electrolytes, Mn_3_O_4_ tends to form at the LiMn_2_O_4_ grain surface upon charging to 4.3 V vs Li/Li^+^, leading to partial irreversibility and cracking.^8,13^ A recent work by Zhao et al. has shown that Mn_3_O_4_ nanowalls can be in situ electrochemically oxidized in Li_2_SO_4_ electrolyte to form Li_4_Mn_5_O_12_, a transformation with potential applications in supercapacitors.^50^ Our TERS and macro-Raman spectroscopy measurements do not conclusively reveal the formation of Li_4_Mn_5_O_12_ or any other Mn-based polymorph upon cycling that could potentially correlate with the observed Mn_3_O_4_ dissolution. However, the stability in the charge–discharge profiles suggests that this dissolution at the surface does not compromise the electrochemically active LMO electrode performance. It may even serve to protect the electrode from potential Mn^2+^ dissolution. We emphasize that our findings suggest a possible surface-related dissolution of Mn_3_O_4_, supported by indirect evidence, and acknowledge the complexities involved in directly characterizing the electrolyte in our experimental setup.

Our findings regarding the presence of ionic species from the electrolyte anchored at the grain boundaries and surface not only contribute to unraveling the complexities of grain boundary chemistry but also align with recent efforts to incorporate lithium sulfate protective coatings in high-voltage cathodes, as documented in the literature.^51,52^ These protective coatings can serve as a proactive shield against the evolving nature of grain boundaries, potentially mitigating the adverse effects of electrolyte interactions. Understanding the intricate interplay between grain boundaries and electrolytes is a pivotal step toward designing cathode materials that can perform well in the long term. Our work also holds broader implications for the stability of cathodes in aqueous electrolytes. For that, we argue that the presence of subnanometer pores and cationic vacancies can potentially help us to mitigate the volume expansions in all-solid electrodes. These insights are not only pertinent to aqueous systems but also offer valuable lessons for enhancing the stability of cathodes in organic electrolytes and solid-state composites, where the behavior of grain boundaries and their interactions remain critical yet are less explored. To gain deeper insights into the dynamic processes occurring during the early stages of cycling, future studies may benefit from developing in situ methods to correlate specific topographic features with the different stages of charge and discharge cycles, thereby enhancing our understanding of the transitions between LiMn_2_O_4_, Li_0.5_Mn_2_O_4_, and λ-MnO_2_ phases, as well as the location and progressive adsorption of sulfates at the surface. Future work will need to address what happens at grain boundaries in more technologically relevant organic electrolytes. At the moment, we are unable to perform such experiments reliably since our equipment is not in a protective atmosphere.

## Conclusions

Our research provides valuable structural and chemical insights across a spectrum of length scales for the defect evolution with cycling in LMO cathodes. Beginning at the subnanometer scale, we have identified vacancy-like defects and their clusters, with the latter emerging as the most prevalent defect within our films. These defects appear to grow in size with cycling, possibly due to the dissolution of Mn^2+^ ions, introducing a degree of disorder into the films. Additionally, we have discerned the presence of two distinct families of subnanometer pores; notably, the larger pores (τ_4_) appear to reach a saturation point with cycling, hinting either at a complete fill-up of part of the pores in a gradual manner with electrolyte species or pore coalescence with cycling.

Zooming out to the nanoscale, our TERS measurements unveil that unlike in organic electrolytes, Mn_3_O_4_ does not form at the surface during cycling in aqueous electrolytes. Instead, it undergoes dissolution and likely electrochemical transformation into another Mn-based phase, which remarkably maintains overall performance and stability. TERS also highlights the preferential adsorption of SO_4_^2–^ species at grain boundaries with their bond strength appearing to intensify during cycling. In addition to the diminishing Mn_3_O_4_ signal, TERS and macro-Raman spectroscopy suggest an increase in disorder within the electrochemically active Raman modes, aligning seamlessly with the observed enlargement of defects at the subnanometer scale. Notably, this marks the first successful utilization of TERS to investigate grain boundary effects in battery materials, revealing the strong bonding of electrolyte-derived species to the grain boundary core and its surroundings.

Expanding our observations to the macroscopic scale, macro-Raman spectroscopy provides robust confirmation of the increased disorder within the electrochemically active phases. Importantly, macro-Raman spectroscopy maintains the overall intensity of the Mn_3_O_4_ impurities peaks. This finding validates our earlier TERS discovery that Mn_3_O_4_ impurities primarily dissolve at the surface. Notably, weak points, particularly grain boundaries, appear to be safeguarded by the absorption of sulfate ions.

Our study advances our understanding of this cathode behavior in aqueous electrolytes, holding great promise for the development of more sustainable and stable electrode materials. We hope this knowledge does not only pertain to aqueous systems but also extends toward enhancing the stability of cathodes in organic electrolytes. The dynamic nature of grain boundaries and their interactions with electrolytes must be taken into account in future protective coating designs.

## Experimental Section

### Thin-Film Fabrication

Commercially available ceramic targets of LiMn_2_O_4_ and Li_2_O (CODEX) were employed for the Larga-Area Pulsed Laser Deposition (LA PLD-5000, PVD Products), equipped with a Coherent (Lambda Physik) COMPex Pro 205 KrF excimer laser (λ = 248 nm). Both targets were ablated sequentially following a multilayer approach, as previously described.^37^ The multilayer sequence involved a consistent ratio of ablation pulses, specifically 4:3 between LMO (800 pulses per cycle) and Li_2_O (600 pulses per cycle). These depositions were conducted at 650 °C and 20 mTorr p_O2_, at a target-substrate distance of 90 mm, constant rotation and laser fluence of 1.3 J·cm^–2^ inside the chamber. The substrates were Pt (80 nm)/Ti (10 nm)/ Si_3_N_4_ (300 nm)/ SiO_2_ (100 nm)/Si (0.5 mm) chips of 1 × 1 cm^2^, fabricated at the Institute of Microelectronics of Barcelona (IMB-CNM-CSIC). Neither the TERS nor the PALS measurements require any sample preparation and could be directly measured after electrochemistry experiments.

### Structural Characterization

Scanning electron microscopy (SEM) was performed at IREC with a Zeiss Auriga instrument (30 kV Gemini FESEM column and an in-lens detector). X-ray diffraction was performed thanks to an XRD Bruker-D8 Advance diffractometer in the θ–2θ configuration between 10 and 60° (step size of 0.01°). The region around the main reflection of Pt was avoided during the measurement to protect the detector.

### Raman Characterization

TERS measurements were performed on an Xplora Nano (HORIBA), with a 632 nm laser with p-polarization (parallel to the tip axis), Au-coated OMNI TERS tips, 100× objective, and a laser power of ∼3 mW. Maps were acquired with a resolution of 10 nm per pixel to build 500 × 500 nm maps, plotted using a 2 px weight average. Acquisition times of 5 s per spectra and a 1200 g·mm^–1^ grating were used. The protocol to ensure the TERS signal consists of checking the TERS signal on a carbon nanotube sample test before and after the experiment. In addition, TERS is demonstrated in our films; the reader is referred to the Supporting Information, Section III, for more details about our TERS configuration. For the macro-Raman spectroscopy measurements, Raman scattering spectra were measured using an iHR320 Horiba Jobin-Yvon monochromator coupled with i-DUS CCD from Andor. NIR solid-state laser (785 nm, ∼ 80 W/cm^2^) was used as an excitation source. A high working distance objective, an Olympus LMPLFLN 20X was employed, while the spot size was empirically defined. The measurements were performed in backscattering configuration through the specific probes developed in IREC. The spectral position was corrected using monocrystalline Si as a reference by imposing the main Raman peak to 520 cm^–1^. Please note that the thick Pt layer reflects the laser entirely and avoids any signal from the Si substrate. TERS measurements were performed using a 633 nm excitation wavelength. However, when these measurements were attempted with a macro-Raman setup, a high fluorescence background was observed, impeding reliable assessment of secondary phases or their changes. Subsequently, we transitioned to a 785 nm laser for our Raman measurements, as similar Raman responses were anticipated in our films. This decision was based on the resonance present in the λ-MnO_2_ phase near the 532 nm excitation wavelength, which could potentially amplify its response, masking other spectral information.^44^ Near-field signal of every tip and experiment is checked on a test sample of carbon nanotubes. Then, the TERS enhancement is double-checked again before and after each map. Our setup is a room with controlled temperature that minimizes thermal and mechanical drift from the AFM stage and the objective scanner.

### Variable Energy Positron Annihilation Lifetime Spectroscopy

VEPALS was carried out at the Monoenergetic Positron Source (MePS) beamline at HZDR (Germany)^53^ using a digital lifetime CrBr3 scintillator detector. This was coupled to a Hamamatsu R13089-100 PMT μ-metal shielded and housed inside a Au solid casing. We used homemade software employing a SPDevices ADQ14DC-2X digitizer with 14-bit vertical and 2GS/s horizontal resolutions.^54^ The time resolution function was decreased to 0.250 ns. The spectra analysis consisted of a resolution function of two Gaussian with distinct intensities and shifts depending on the positron implantation energy, *E*_p_. All spectra contained at least 1 × 10^7^ counts. Typical lifetime spectrum *N*(*t*) is described by *N*(*t*) = Σ_*i*_ (1/τ_*i*_) *I_i_* exp(−*t*/τ_*i*_), where τ_*i*_ and *I_i_* are the positron lifetime and intensity of the *i*th component, respectively (Σ*I_i_* = 1). All the spectra were deconvoluted using a nonlinear least-squares fitting method employed within the fitting software package PALSfit.^55^ It consisted of 5 discrete lifetime components, which directly show localized annihilation at 2 different defect types (sizes; τ_1_ and τ_2_), i.e., small vacancy-like defect and their agglomerations or clusters. third and fourth lifetime components (τ_3_ and τ_4_) are two pore populations of diameters *d*_3_ and *d*_4_. The fifth component (not shown) is residual and originates from orthopositronium annihilation in vacuum and pore networks. The pore size was derived using the Wada and Hyodo shape-free model.^39^ The positron lifetime and its intensity have been probed as a function of positron implantation energy *E*_p_ or equivalently, implantation depth or film thickness. Positrons have been accelerated and monoenergetically implanted into samples in the range between *E*_p_ = 1–12 keV for depth profiling. A mean positron implantation depth was approximated using a simple material density (ρ = 4.02 g·cm^–3^) dependent formula: < *z* ≥ 36/ρ·*E*_p_^1.62^.^56^ The average positron lifetime τ_average_ is defined as τ_average_ = ∑*i* τ_*i*_·*I_i_*.

### Electrochemical Measurements

For electrochemical measurements, we established electrical contacts with the LMO films by accessing the exposed Pt regions at the sample edges, securely embedding them within a durable dual-component resin. This resin was applied thin enough to facilitate mechanically stable TERS and PALS characterizations. The electrochemical measurements were conducted employing an open cell assembly, wherein a substantial portion of the film’s surface remained exposed, facilitated by the resin’s coverage. The exposed surface area typically ranged from 30 to 40 mm^2^. This configuration allowed for the rapid quenching of the electrochemical cell, subsequent immersion of the film in DI water, and fast drying using a nitrogen gun for storage within a protective atmosphere. Some of the films were cycled with a TSC Surface cell (rhd instruments, Germany). The electrochemical experiments encompassed a voltage window spanning from 0.5 to 1.05 V versus Ag/AgCl (3 M KCl), employing a Pt mesh as the counter electrode and 1 M Li_2_SO_4_ electrolyte. The electrochemical measurements on charge–discharge profiles were performed in constant current mode, keeping the same current density of 40 μA·cm^–2^ (∼5C rate).

## Supporting Information Available

Section I: Variable energy positron annihilation lifetime spectroscopy (VEPALS) – as-deposited; Section II: Positron annihilation lifetime spectroscopy as a function of thickness and cycling; Section III: Macro-Raman and tip-enhanced Raman spectroscopy (TERS) characterization (PDF)
