# Supplementary material for: Insights into the LiMn2O4 Cathode Stability in Aqueous Electrolytes
Source: Chem Mater. 2024 Jun 3;36(12):6144–53. doi: 10.1021/acs.chemmater.4c00888 (PMC11209945; doi:10.1021/acs.chemmater.4c00888)
Supplement: Supplementary file 1 — cm4c00888_si_001.pdf [file cm4c00888_si_001.pdf]

SUPPORTING INFORMATION

Insights into the $LiMn_2O_4$ Cathode Stability in Aqueous Electrolyte

Juan Carlos Gonzalez-Rosillo[a*], Maxim Guc[a], Maciej Oskar Liedke[b], Maik Butterling[b], Ahmed G. Attallah[b], Eric Hirschmann[b], Andreas Wagner[b], Victor Izquierdo-Roca[a], Federico Baiutti[a], Alex Morata[a*], Albert Tarancón[a,b,*]

[a] Catalonia Institute for Energy Research (IREC), Jardins de les Dones de Negre 1, Planta 2, 08930, Sant Adrià del Besòs, Barcelona, Spain

[b] Catalan Institution for Research and Advanced Studies (ICREA), Passeig Lluís Companys 23, 08010, Barcelona, Spain

[c] Helmholtz-Zentrum Dresden - Rossendorf, Institute of Radiation Physics, Bautzner Landstraße 400, 01328 Dresden, Germany

*Corresponding authors

SECTION I: Variable Energy Positron Annihilation Lifetime Spectroscopy (VEPALS) – as-deposited

The characterization of the films with VEPALS necessitates the deconvolution of the average positron lifetime into a certain number of components. The spectra analysis required a two Gaussian resolution function, each with different shifts and intensities as a function of the positron implantation energy, E_p. Typical lifetime spectrum N(t) is described by $N(t)=\Sigma_i (1/\tau_i) I_i \exp(-t/\tau_i)$, where τ_i and I_i are the positron lifetime and intensity of the i-th component, respectively ($\Sigma I_i=1$). The deconvolution of the spectra was performed using a non-linear least-squares fitting method employed within the fitting software package PALSfit [1]. It comprised five discrete lifetime components, each directly linked to localized annihilation at two different defect types (sizes; τ_1 and τ_2) representing small vacancy-like defects and their agglomerations (clusters). The 3rd and 4th-lifetime components (τ_3 and τ_4) corresponded to two pore populations with diameters d_3 and d_4. The 5th component (not displayed) arose from ortho-positronium annihilation in vacuum and pore networks. The positron lifetime and its intensity were explored as a function of positron implantation energy E_p, which corresponds to implantation depth or film thickness. Positrons were accelerated and monoenergetically implanted into samples within the range of Ep = 1-12 keV for depth profiling. A mean positron implantation depth was approximated using a simple material density (ρ = 4.02 g·cm^{-3}) dependent formula: $\langle z\rangle=36/\rho \cdot E_p^{1.62}$.[2] The average positron lifetime $\tau_{average}$ is defined as $\tau_{average} = \sum i\ \tau_i \cdot I_i$.

The positron lifetime versus positron implantation energy E_p (related to thickness) shows a rather homogeneous value across the entire thickness (main text, Fig. 1c). Spectra deconvolution unveiled four types of defects: First, small vacancy-like defects and their clusters (τ_1 and τ_2 with relative intensities I_1 and I_2, respectively), Fig. S1a,b. Second, two families of sub-nm pores τ_3 and τ_4 with spherical sizes $d_3 \approx 0.47$ nm and $d_4 \approx 0.75$ nm and intensities I_3 and I_4, respectively, Figure S1c,d. Overall, all these defects show a homogeneous distribution along the film thickness. The comparison of the relative intensities points out the dominance of vacancy-related defects, ($I_1 + I_2 > I_3 + I_4$), indicating the larger presence of vacancies when compared to pores. These kinds of

vacancies have also been observed by PALS in $LiCoO_2$ cathodes and have been ascribed to lithium vacancies (τ_1) and clusters of lithium vacancies (τ_2), respectively.[3,4] However, we would rather be cautious over the assignment of the type of vacancy (lithium vs. manganese vacancies), since similar lifetimes have been reported for Mn vacancies.[5] In addition, the shortest lifetime component, τ1, is essentially convoluted with para-Positronium signal (120 ps), accompanying ortho-Positronium annihilation in pores. The ratio p-Ps to o-Ps is 1:3.[6] The existence of the p-Ps signal contaminates the short lifetime component data, and therefore, we would rather be cautious at this moment about the exact nature of the type of vacancy.

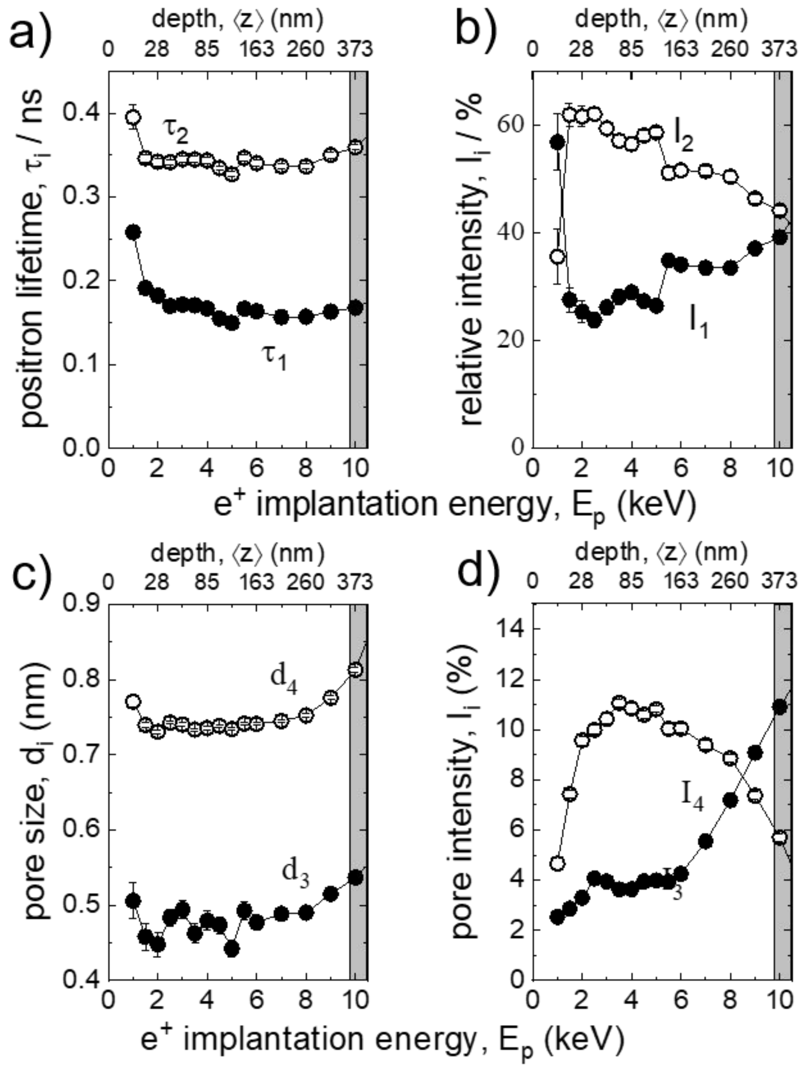

Figure S1. Deconvolution of PALS spectra into the different components. a) positron lifetime for vacancy-related defects, τ_1 and τ_2. b) relative intensities of vacancy-related defects, I_1 and I_2. c) pore size derived from lifetime components τ_3 and τ_4, d_3 and d_4, respectively. d) pore relative intensity, I_3 and I_4.

SECTION II: Positron Annihilation Lifetime Spectroscopy as a function of thickness and cycling

The PALS analysis of the cycled cathode films shows a clear decrease in the average defect size with cycling, Fig. S2a. The first 100 nm (1/3 of the sample) seems to be already stable after 100 cycles, but deeper on the film shows a further decrease in average defect size at 300 cycles. At first glance, the lifetime components' weight on the average defect size, Fig. S2b-e, it is evident that its decrease with cycling is largely

affected by the changes in the sub-nm pores families, τ_3 and τ_4. The density (relative intensity) of the larger pores, I_4, decreases from ~15% to ~6%, essentially vanishing in deeper parts of the film. The size of this pore family suffers a slight increase while the d_3 decreases in size across the thickness after 300 cycles. These observations could indicate that both families of pores are being filled with the electrolyte or pores coalescence.

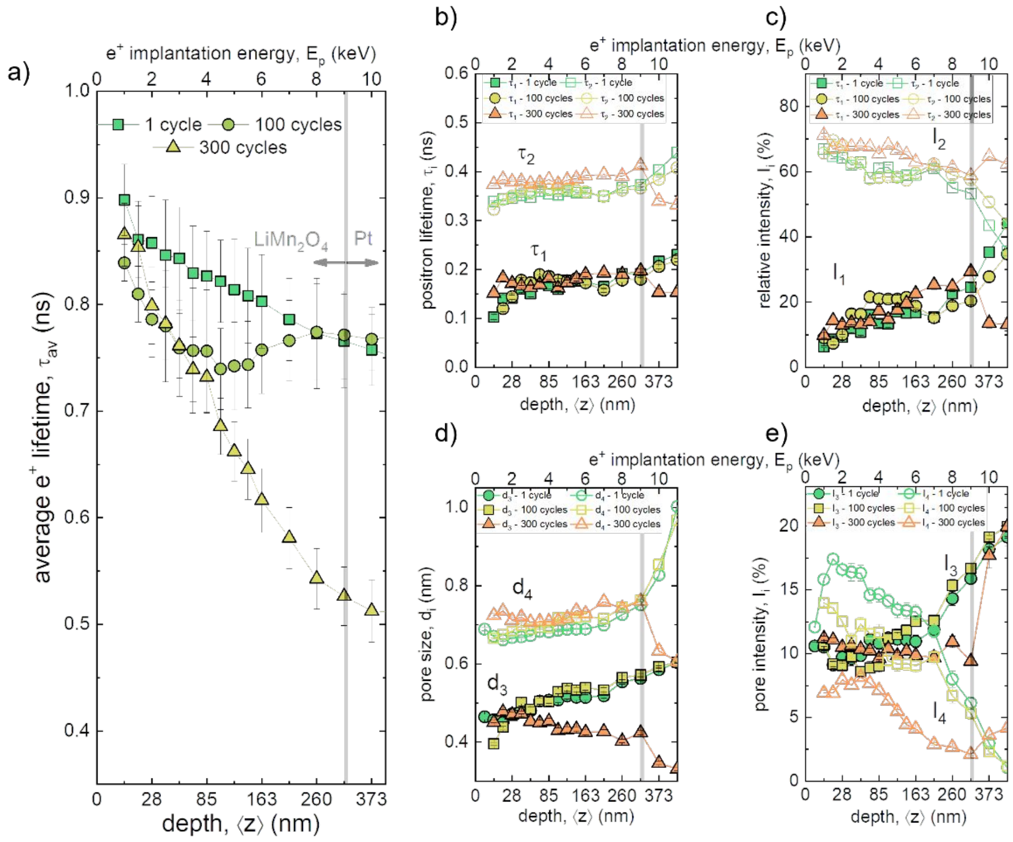

Figure S2: PALS as a function of cycling.a) Positron average lifetime as a function of implantation energy. B) Positron lifetime components for vacancy-like defects (T1) and their clusters (T2). C) Relative intensity of vacancy-like defects (I1) and their clusters (I2), d) Pore diameter for the families of small (d3) and large (d4) pores. e) Relative intensities of the small (I3) and large (I4) families of pores.

The vacancy-like defects (I_1) and their clusters (I_2) do not seem to change their density much with cycling. Regarding their size, vacancy-like defects suffer a slight increase with cycling (more pronounced close to the surface), while the vacancy clusters are enlarged only after 300 cycles across the entire thickness. We hypothesize that these small increases in vacancy-like defects are indicators of the good stability over cycling of the films and might be related to a certain loss in crystallinity and Mn dissolution, as discussed in the main text.

Overall, the PALS analysis of samples subjected to different numbers of cycles revealed a decrease in the average defect size with cycling, primarily attributed to the changes in sub-nm pores families.

SECTION III: Macro-Raman and Tip-Enhanced Raman Spectroscopy (TERS) characterization

Fundamentals and enhancement factor analysis

Testing the limits of TERS capabilities often involves the use of ideal systems, such as our sample test consisting of graphene and CNTs on an Au-coated substrate. This setup serves as a reliable benchmark to determine whether a tip is TERS-active or not. However, when dealing with more realistic environments, like our 300 nm thick films on top of a Pt-coated substrate, the situation becomes more complex. Firstly, due to the limited coupling effect between a metallic substrate and the tip due to the thickness of our films, TERS signal enhancement primarily results from the so-called lightning-rod effect, rather than from the localized surface plasmon resonance.[7] Moreover, LMO exhibits a notably low Raman signal compared to more Raman-friendly materials like organic compounds or silicon, often necessitating slightly higher laser powers to access distinguishable signals. Consequently, this leads to an increased collection of background signals when the laser illuminates the tip apex.

Nonetheless, TERS amplification is consistently observed whether the tip is in contact with the film or retracted from it, Fig. S3. In this context, when examining non-ideal samples using TERS, we typically achieve modest enhancement factors (EFs) around 2.5. Despite this moderate enhancement, the acquired data remains sufficiently sensitive to detect variations in the relative intensities of Raman peaks across the scanned areas with confidence. This fundamental aspect is crucial for the visualization of our TERS results, where different colors correspond to regions where specific peaks are more pronounced across the scanned area.

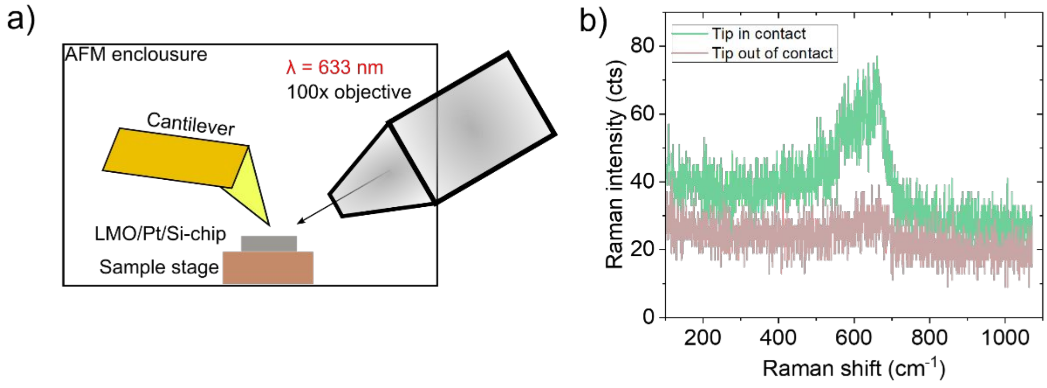

Figure S3 a) Sketch of the TERS setup. b)Comparison of Raman Spectra acquired with the tip in contact and with the tip out of contact. Both spectra were acquired with the same conditions, 5 s per spectra:

Spectra fitting and peak assignment

For the peak fitting, the baseline was removed from the spectra and normalized them to the Mn_3O_4 A_{1g} mode, which is the easiest to identify across the different samples. Let us also state that for all the fittings, Lorentzian functions were used and χ^2 values were in the range 0.05 – 0.5. The fittings are shown in Fig. 4 and the parameters extracted from the fittings are shown in table I.

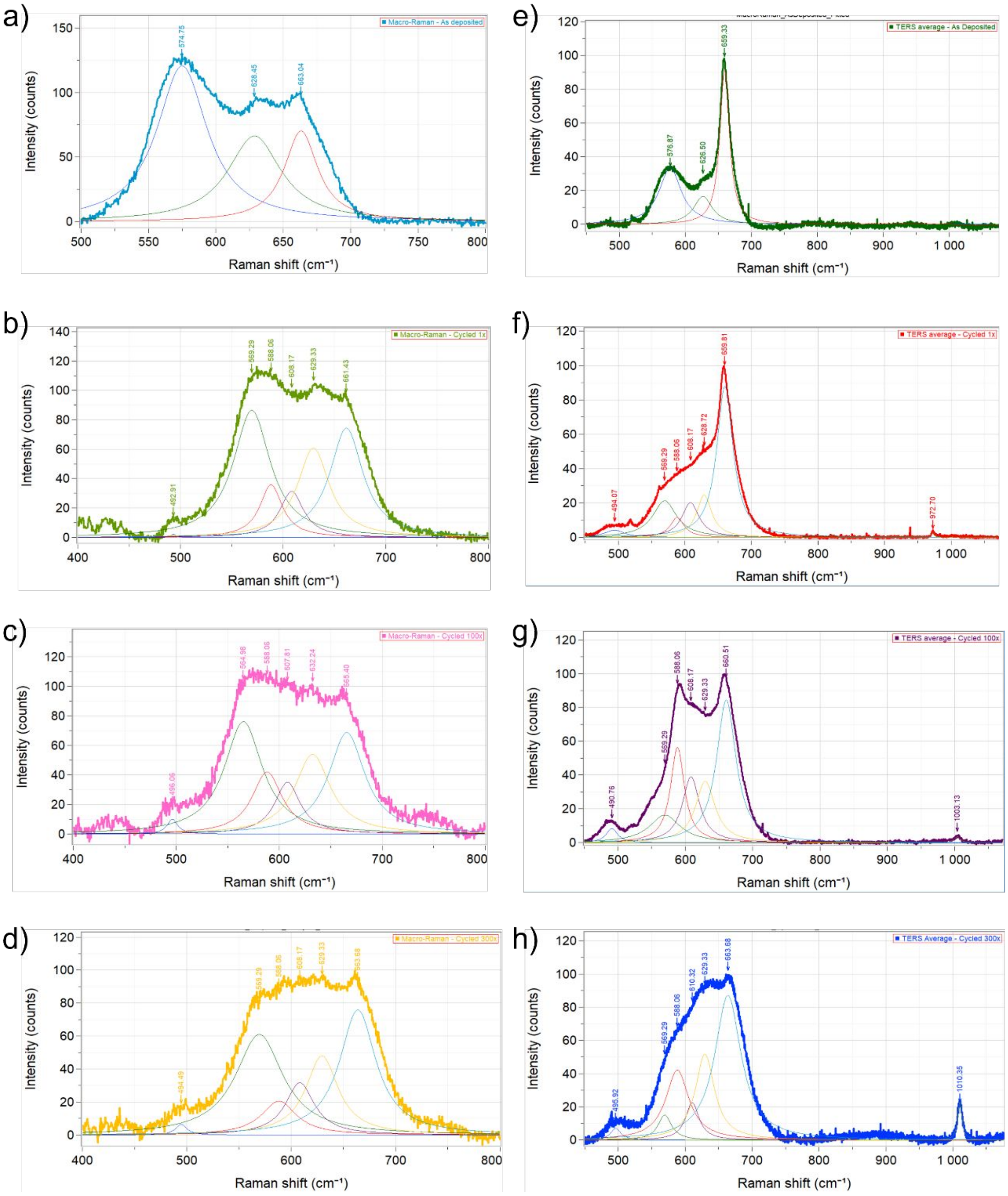

Figure S4: Fittings of the normalized spectra. a), b), c) and d) are the Macro-Raman fittings for the as-deposited, 1, 100 and 300 cycled films, respectively. e), f), g) and h) are the average TERS spectra for the as-deposited, 1, 100 and 300 cycled films, respectively

In the as-deposited state, and supported by our XRD data, only $LiMn_2O_4$ and Mn_3O_4 peaks are found in the spectral region of interest. This means 3 Raman bands, corresponding to The F_{2g} and A_{1g} modes of $LiMn_2O_4$ and the A_{1g} mode of Mn_3O_4. Comparing the as-deposited state by macro-Raman and TERS exposes the large difference between the relative intensity of the Mn_3O_4 peak and the $LiMn_2O_4$ phases. This is strong evidence supporting the larger presence of Mn_3O_4 at the surface of the films in comparison to the bulk. It is important to keep in mind that the Raman signal of Mn_3O_4 is way larger than of $LiMn_2O_4$, especially when using larger wavelengths lasers. In our XRD, it is clear that the films are largely dominated by the LMO phase (by a factor of 10, Fig. 1b). For the cycled films, however, more phases were identified and therefore, more peaks were needed for the fitting. We identified peaks corresponding to $LiMn_2O_4$,

$Li_{0.5}Mn_2O_4$, λ-MnO_2, Mn_3O_4 and the sulfate-related vibrations. Peak assignments are done based on references [8,9].

		Macro Raman				TERS				
		As deposited	Cycled 1x	Cycled 100x	Cycled 300x	As deposited	Cycled 1x	Cycled 100x	Cycled 300x	Peak assignment
Peak 1	Peak position	..	492.908	496.057	494.492	--	494.068	490.765	495.919	
	Peak Amplitude	..	2.37576	10.0601	6.43638	--	4.18936	8.45738	7.08024	
	Peak Width	..	5	12.587	12.937	--	36.281	20.6044	18.8431	
	Area	..	18.4512	193.456	127.071	--	206.82	250.494	194.93	T2g - λ-MnO_2
Peak 2	Peak position	574.753	569.289	564.975	569.289	576.865	569.289	569.289	569.289	
	Peak Amplitude	120.718	86.5023	76.2087	61.0813	33.6107	21.3123	16.4021	15.0827	
	Peak Width	45.5154	44.0148	45.0794	55.3667	40.6609	43.6665	55.5222	24.704	
	Area	7544.59	5553.64	4999.06	4836.39	2010.12	1357.51	1300.99	561.487	T2g - $LiMn_2O_4$
Peak 3	Peak position	--	588.058	588.063	588.058	--	588.058	588.058	588.058	
	Peak Amplitude	--	35.8962	42.0979	20.6197	--	11.5879	56.5808	42.1671	
	Peak Width	--	26.88	35.7836	40.6211	--	25.4758	24.8322	42.1816	
	Area	--	1450.7	2231.44	1230.66	--	446.262	2125.82	2620.49	A1g - λ-MnO_2
Peak 4	Peak position	--	608.17	607.806	608.17	--	608.17	608.17	610.316	
	Peak Amplitude	--	31.4106	35.0484	31.7381	--	20.2485	39.0937	22.6273	
	Peak Width	--	28.3091	30.4059	33.7707	--	31.3536	30.9641	24.5999	
	Area	--	1333.85	1593.02	1593.22	--	955.169	1822.04	845.659	A1g - $Li_{0.5}Mn_2O_4$
Peak 5	Peak position	628.453	629.332	632.236	629.332	626.504	628.722	629.332	629.332	
	Peak Amplitude	66.4365	60.883	54.0033	48.069	16.7253	24.6052	36.456	51.8264	
	Peak Width	48.4225	37.7572	42.1085	37.8427	34.5649	27.4658	35.3046	34.8849	
	Area	4528.7	3389.95	3327.33	2682.14	868.711	1025.17	1932.75	2716.87	A1g - $LiMn_2O_4$
Peak 6	Peak position	663.037	661.431	665.4	663.677	659.328	659.81	660.507	663.677	
	Peak Amplitude	70.2693	74.4587	68.7667	76.0496	91.4351	87.956	84.5138	87.2411	
	Peak Width	30.274	39.2087	42.6195	42.4554	17.5407	29.8403	35.0415	47.8703	
	Area	3126.25	4271.55	4256.35	4692.75	2468.77	3982.06	4465.54	6205.69	A1g - Mn_3O_4
Peak 7	Peak position	--	--	--	--	--	972.704	1003.13	1010.35	
	Peak Amplitude	--	--	--	--	--	3.19537	3.44605	23.1359	
	Peak Width	--	--	--	--	--	5.2022	11.0962	7.55793	
	Area	..	--	--	--	--	25.849	58.2966	269.01	sulfate based

Table I: Peak parameters extracted from the fittings.

These fittings also help to understand whether the Mn_3O_4 band remains unaffected by cycling and whether the electrochemically active phases decreased their amplitude. Fig. S5a shows the amplitudes of the $LiMn_2O_4$ and the Mn_3O_4 bands in the as-deposited state and after the different cycles. It is immediately seen that the amplitudes of the $LiMn_2O_4$ A_{1g} and F_{2g} bands decrease with cycling while the Mn_3O_4 remains more or less constant in the cycling range explored. It is worth emphasizing that the comparison between the macro-Raman and TERS measurements show contrasting behaviors in the A_{1g} $LiMn_2O_4$/Mn_3O_4 peak ratio, indicating potential surface-related Mn_3O_4 degradation and dissolution. (Fig. S5b).

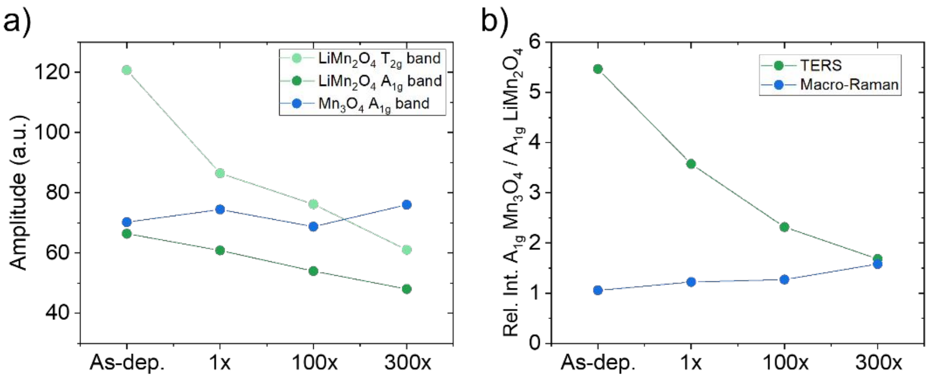

Figure S5: a) Macro-Raman amplitudes of the $LiMn_2O_4$ and Mn_3O_4 bands, extracted from the fittings. b) Relative intensity of the Mn3O4 and LiMn2O4 bands for macro-Raman and TERS as a function of cycling.

Regarding the analysis of the sulfate-based band observed by TERS, Fig. S6 shows the different parameters (peak amplitude, peak area and peak position) varying with cycling. It is clear that the peak amplitude changes drastically after 300 cycles. However, the changes are clearer in the peak area and peak position. The peak area increases with

cycling, suggesting an increasing presence at the surface upon cycling. In addition, the progressive shift in the peak position, from 972 cm^{-1} to 1010 cm^{-1} after 300 cycles suggests a change in the chemical bond itself. Sulfate ion vibrations are known to be in the range 970-980 cm^{-1}, so it seems reasonable to deduce that, at the first stages of cycling sulfate ions are adsorbed at grain and grain boundaries thanks to the positively charged space charge layers around the core of the grain boundaries, as described in the manuscript. With cycling, however, the shift of the sulfate band up to 1010 cm^{-1} suggests that these bonds are shorter and stronger than the adsorbed ions that are observed after 1 cycle. It could be possible that the resulting peak is centered around 1010 cm^{-1}, correlates well with the main Raman band of solid Li_2SO_4 (see, for instance, https://spectrabase.com/spectrum/7nHAJAZ9o8T). Please notice also, as described in the manuscript, that the family of larger sub-nm pores also collapsed with cycling, suggesting that these pores might be filling progressively with cycling. We suggest that, upon cycling, lithium sulfate might be formed at the surface and grain boundaries and collapse part of the sub-nm pores of the film. This would be kind of a favorable SEI.

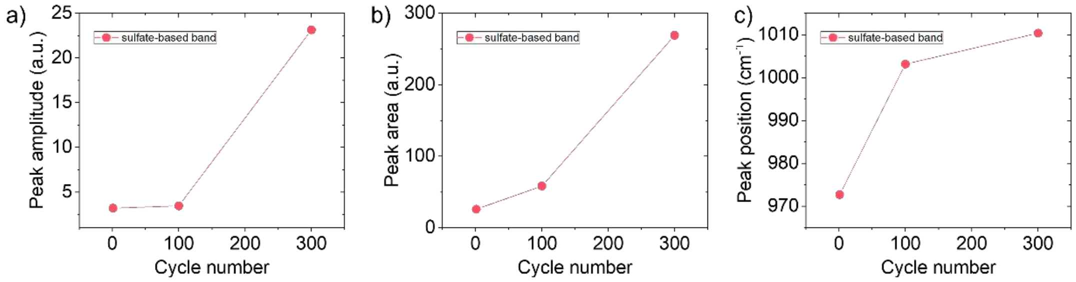

Figure S6: Analysis of the sulfate-based vibrations observed by TERS as a function of cycling, extracted from the fittings. a) Peak amplitude. b) Peak area. c) Peak position.

TERS mapping

For the TERS mapping, we proceed in the same manner as described for the macro-Raman and TERS average spectra. At each pixel of the map, spikes and baseline are removed, spectra normalized and peaks fitted. Then, each peak is plotted separately to create a visualization of the relative intensity of the selected band across the map. For all the maps, the same criteria has been used to plot the relative intensities: the intensity data were normalized to a maximum intensity of 1, and plotted in the range from 1 – 0.5. This ensures a consistent intensity scale across all maps, allowing for direct comparison and correlation of relative peak intensities and topography in the TERS mapping overlay. Each pixel has an average factor of 1.4 in the XY plane. This is due to the use of 10 nm step during the TERS measurement and the larger size of the tip (~20 nm). This small average aids in the identification of clusters in the maps without compromising the spatial resolution.

For the direct correlation with particular topographic features, we use the topography image that is obtained simultaneously with the spectroscopy. In other words, each of the pixels represented in the topography map shows the location where the spectra were recorded. In this way, our overlayed maps avoid errors due to thermal drift of misalignment with a topography obtained before or after the spectroscopy map. The overlay is performed using GIMP software. We added here a larger size panels with all the TERS maps and overlays of the manuscript.

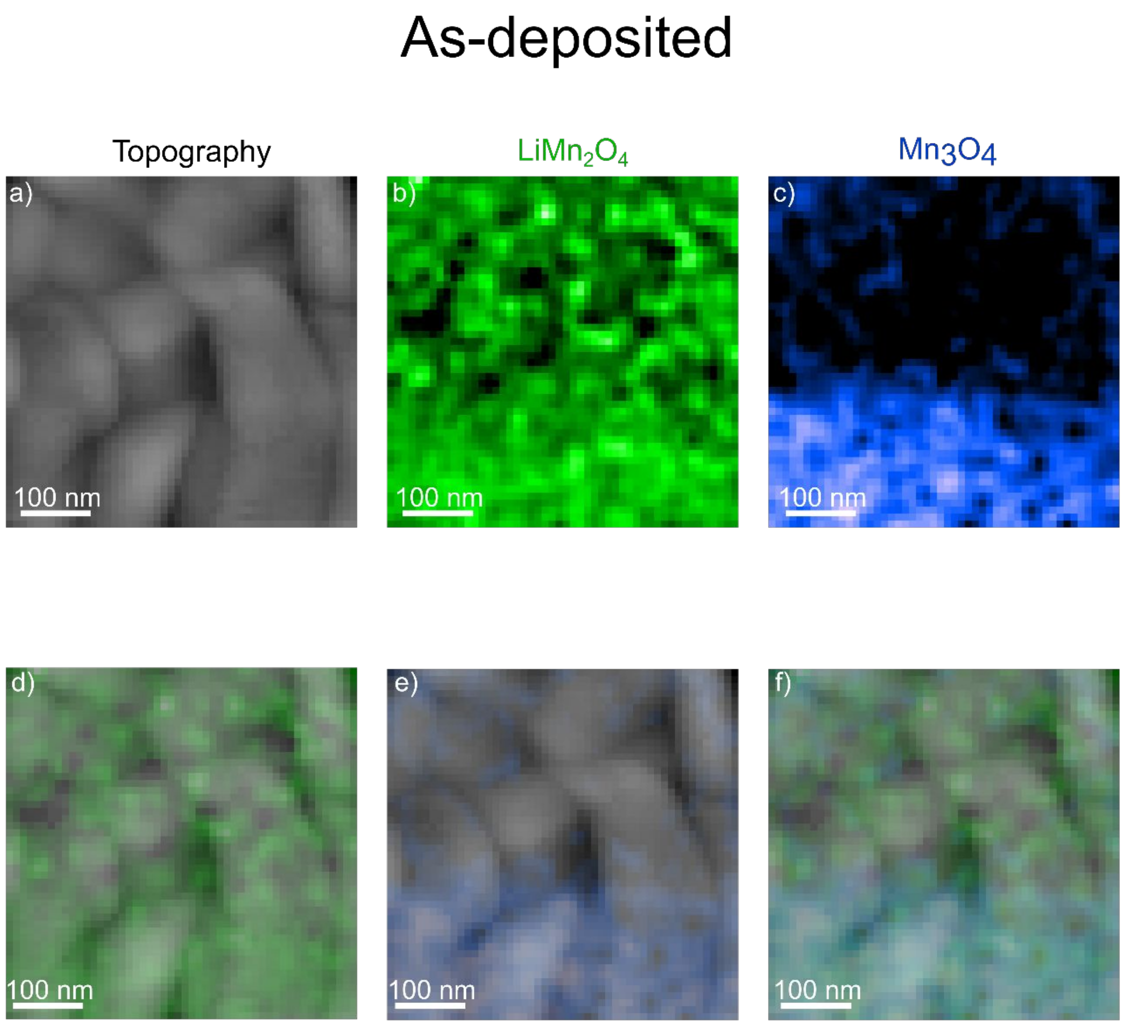

Figure S7: a) Topography obtained during the TERS mapping for the as-deposited state. TERS maps: b) Green represents the $LiMn_2O_4$ phase and c) blue the Mn_3O_4 phase. Overlays of d) Topography and $LiMn_2O_4$ phase, e) topography and Mn_3O_4 and f) topography and both phases. All panels are 500 x 500 nm^2.

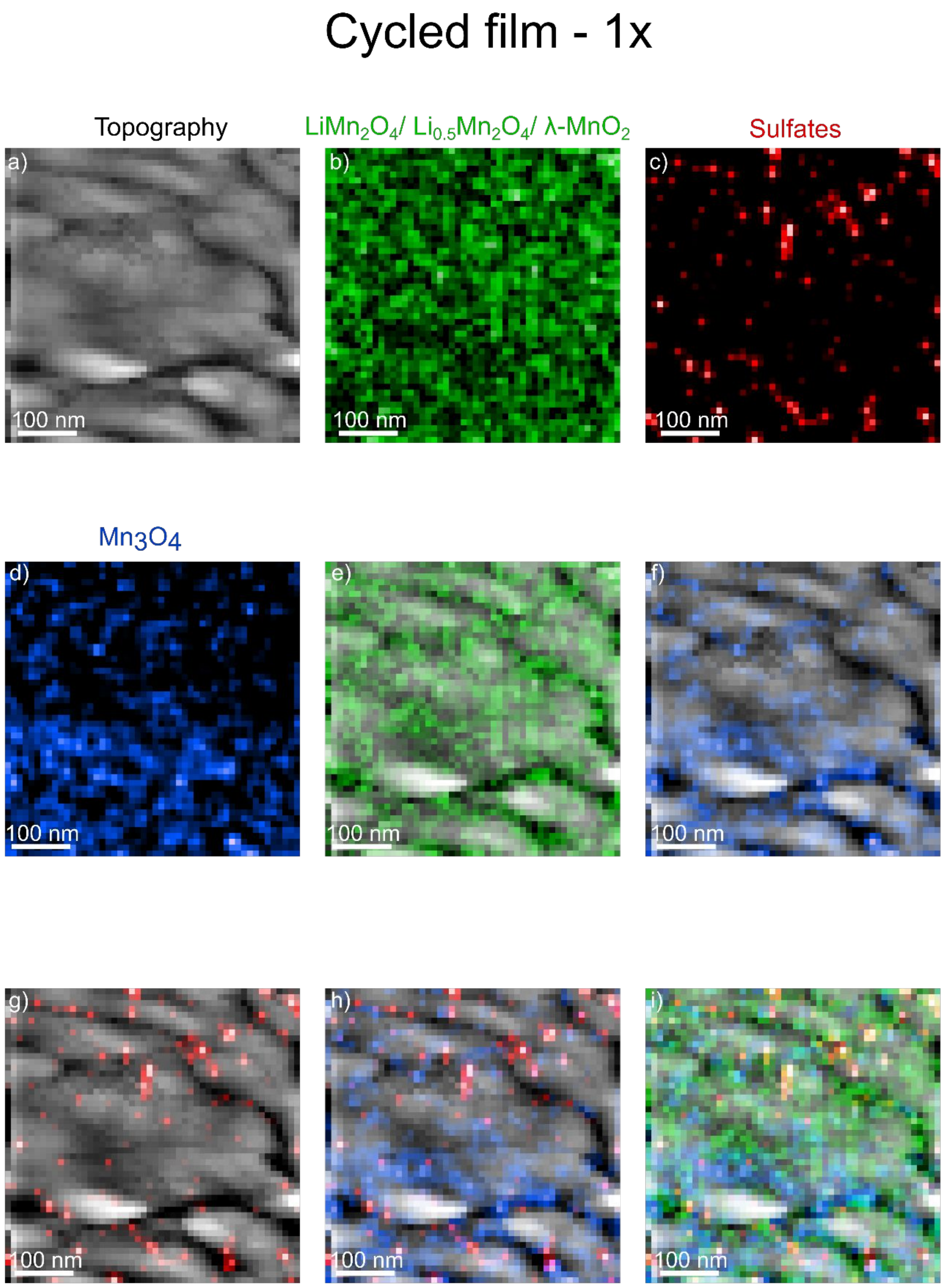

Figure S8: a) Topography obtained during the TERS mapping after 1 cycle. TERS maps: b) Green represents the $LiMn_2O_4$ phase, c) red represents the sulfate band and d) blue the Mn_3O_4 phase. Overlays of e) topography and $LiMn_2O_4$ phase, f) topography and Mn_3O_4, and g) topography and sulfates, h) topography and sulfates and Mn_3O_4 f) topography and both phases. All panels are 500 x 500 nm^2.

Cycled film - 100x

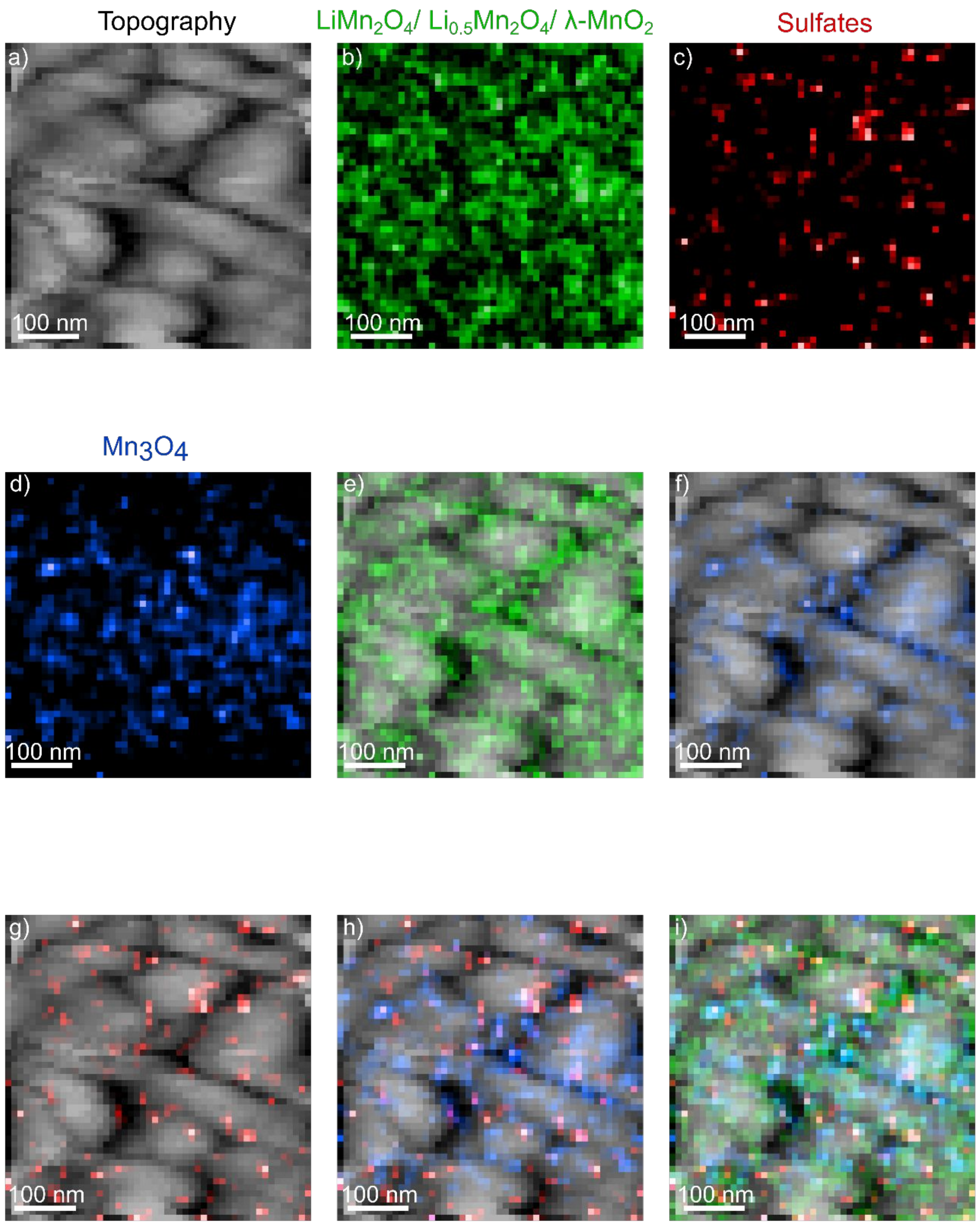

Figure S9: a) Topography obtained during the TERS mapping after 100 cycles. TERS maps: b) Green represents the $LiMn_2O_4$ *phase, c) red represents the sulfate band and d) blue the* Mn_3O_4 *phase. Overlays of e) topography and* $LiMn_2O_4$ *phase, f) topography and* Mn_3O_4*, and g) topography and sulfates, h) topography and sulfates and* Mn_3O_4 *f) topography and both phases. All panels are 500 x 500* nm^2.

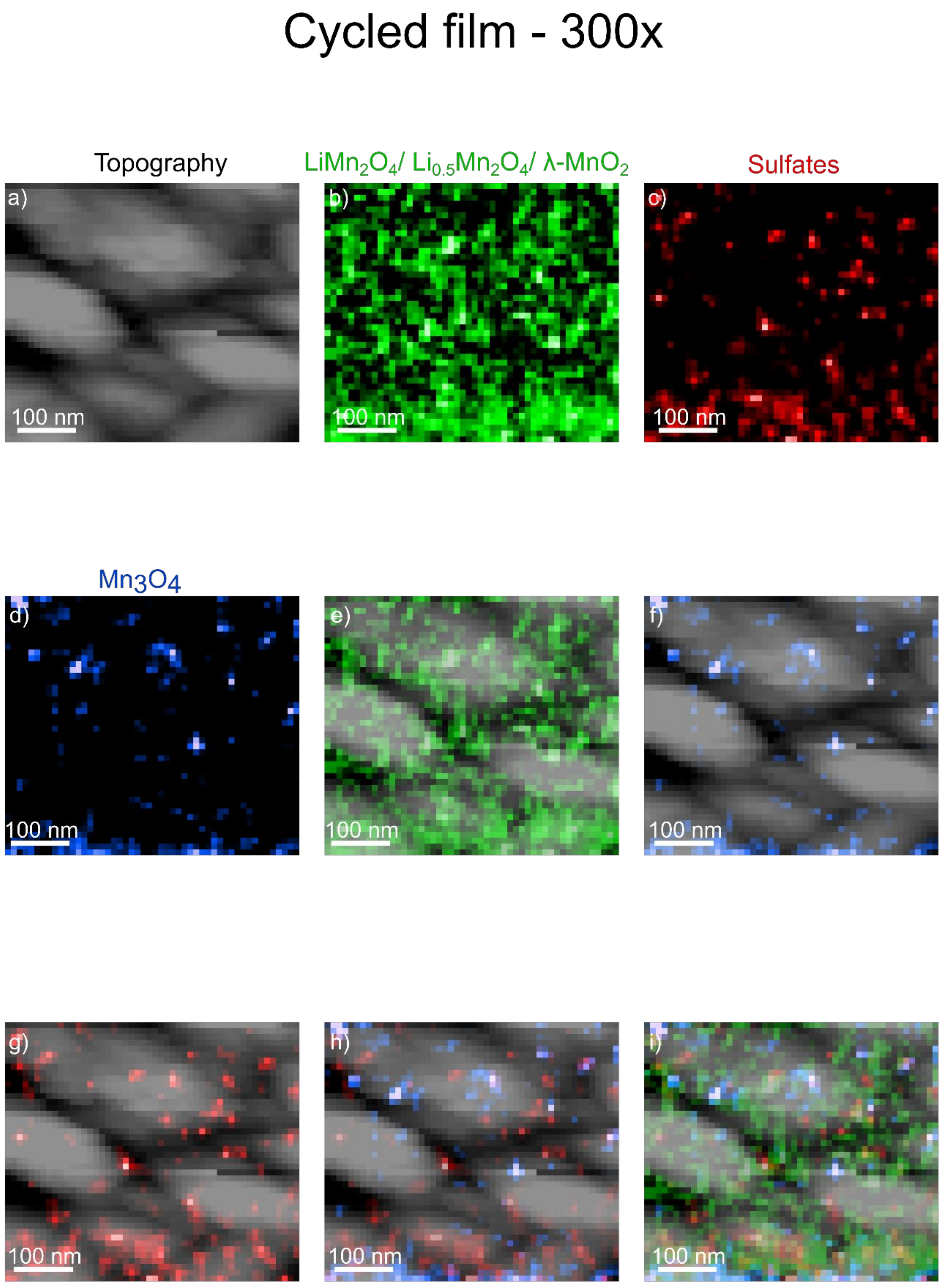

Figure S10: a) Topography obtained during the TERS mapping after 300 cycles. TERS maps: b) Green represents the $LiMn_2O_4$ phase, c) red represents the sulfate band and d) blue the Mn_3O_4 phase. Overlays of e) topography and $LiMn_2O_4$ phase, f) topography and Mn_3O_4, and g) topography and sulfates, h) topography and sulfates and Mn_3O_4 f) topography and both phases. All panels are 500 x 440 nm^2.

REFERENCES

[1] J. V. Olsen, P. Kirkegaard, N.J. Pedersen, M. Eldrup, PALSfit: A new program for the evaluation of positron lifetime spectra, Phys. Status Solidi. 4 (2007) 4004–4006. https://doi.org/10.1002/pssc.200675868.

[2] J. Dryzek, P. Horodek, GEANT4 simulation of slow positron beam implantation profiles, Nucl. Instruments Methods Phys. Res. Sect. B Beam Interact. with Mater. Atoms. 266 (2008) 4000–4009. https://doi.org/10.1016/j.nimb.2008.06.033.

[3] G. Pagot, V. Toso, B. Barbiellini, R. Ferragut, V. Di Noto, Positron annihilation spectroscopy as a diagnostic tool for the study of licoo2 cathode of lithium-ion batteries, Condens. Matter. 6 (2021) 1–11. https://doi.org/10.3390/condmat6030028.

[4] J. Nokelainen, B. Barbiellini, J. Kuriplach, S. Eijt, R. Ferragut, X. Li, V. Kothalawala, K. Suzuki, H. Sakurai, H. Hafiz, K. Pussi, F. Keshavarz, A. Bansil, Identifying Redox Orbitals and Defects in Lithium-Ion Cathodes with Compton Scattering and Positron Annihilation Spectroscopies: A Review, Condens. Matter. 7 (2022) 1–18. https://doi.org/10.3390/condmat7030047.

[5] V.P. Dinh, T.A. Luu, K. Siemek, D.P. Kozlenko, K.H. Le, N.T. Dang, T. Van Nguyen, N. Le Phuc, T.D. Tran, P.T. Phan, S.T. Lo, K.A.T. Hoang, T.K. Dinh, N.T. Luong, N.C. Le, N.T. Nguyen, T.H. Ho, X.D. Tran, P.D. Tran, H.Q. Nguyen, P.D. Tran, Crystallization Pathways and Evolution of Morphologies and Structural Defects of α-MnO2under Air Annealing, Langmuir. 38 (2022) 15604–15613. https://doi.org/10.1021/acs.langmuir.2c02237.

[6] R. (Reinhard) Krause-Rehberg, Positron annihilation in semiconductors : defect studies, Springer, Berlin ;, 1999.

[7] T.X. Huang, S.C. Huang, M.H. Li, Z.C. Zeng, X. Wang, B. Ren, Tip-enhanced Raman spectroscopy: Tip-related issues, Anal. Bioanal. Chem. 407 (2015) 8177–8195. https://doi.org/10.1007/s00216-015-8968-8.

[8] C.M. Julien, M. Massot, Lattice vibrations of materials for lithium rechargeable batteries III. Lithium manganese oxides, Mater. Sci. Eng. B. 100 (2003) 69–78. https://doi.org/10.1016/S0921-5107(03)00077-1.

[9] N. Kuwata, Y. Matsuda, T. Okawa, G. Hasegawa, O. Kamishima, J. Kawamura, Ion dynamics of the Li Mn2O4 cathode in thin-film solid-state batteries revealed by in situ Raman spectroscopy, Solid State Ionics. 380 (2022) 115925. https://doi.org/10.1016/j.ssi.2022.115925.
